# *Arhgap29* Deficiency Directly Leads to Systemic and Craniofacial Skeletal Abnormalities

**DOI:** 10.3390/ijms26104647

**Published:** 2025-05-13

**Authors:** Beibei Zhang, Xiaoyun Pan, Dandan Chi, Yumeng Wang, Wenyan Ruan, Jian Ma, Xiaohong Duan, Yongqing Huang

**Affiliations:** 1 Ningxia Key Laboratory of Oral Disease Research, Ningxia Key Laboratory of Craniomaxillofacial Deformities Research, School of Stomatology, Ningxia Medical University, Yinchuan 750004, China; zhang@nxmu.edu.cn (B.Z.); 13995177028@163.com (X.P.); dan13024350054@163.com (D.C.); wym8916@163.com (Y.W.); majia19880210@126.com (J.M.); 2 State Key Laboratory of Oral & Maxillofacial Reconstruction and Regeneration, National Clinical Research Center for Oral Diseases, Shaanxi Key Laboratory of Stomatology, Department of Oral Biology, Clinic of Oral Rare and Genetic Diseases, School of Stomatology, The Fourth Military Medical University, Xi’an 710032, China; rwy12345679@163.com

**Keywords:** *Arhgap29*, syndromic cleft lip and palate (SCL/P), bone, ectrodactyly

## Abstract

The *Arhgap29* gene encodes Rho-GTPase-activating protein 29 (*Arhgap29*), which plays a crucial role in embryonic tissue development. Mutations in the *Arhgap29* gene are significantly associated with non-syndromic cleft lip and palate (NSCL/P). Our study demonstrated that the deletion of *Arhgap29* leads to syndromic cleft lip and palate (SCL/P) characteristics in mice, where, in addition to cleft palate, the mice exhibit craniofacial and systemic skeletal abnormalities. However, the mechanisms underlying these skeletal abnormalities remain unclear. Through micro-CT imaging, histological analysis, and transcriptomic methods, we discovered that the knockout of *Arhgap29* delays the fusion of Meckel’s cartilage, widens cranial sutures, reduces bone quality, and alters the expression of osteoblasts and osteoclasts in the mandible. Digit defects, including ectrodactyly and impaired endochondral ossification, were also observed. Immunohistochemical analysis demonstrated the expression of *Arhgap29* in both osteoblasts and osteoclasts, indicating its dual role in maintaining matrix homeostasis and regulating bone resorption equilibrium. Transcriptomic analysis revealed disrupted calcium and MAPK signaling pathways, while in vitro studies demonstrated impaired osteogenesis in *Arhgap29*-deficient calvarial cells, mirroring the in vivo defects. Furthermore, spatial transcriptomics linked the loss of *Arhgap29* to defective bone differentiation and protein synthesis. Our findings underscore the critical role of *Arhgap29* in the development of the mandible and digits, suggesting its potential as a pathogenic gene associated with syndromic cleft lip and palate (SCL/P).

## 1. Introduction

Cleft lip with or without cleft palate (CL/P) represents the most prevalent congenital craniofacial malformation, occurring in approximately one in every 700 to 1000 live births [1]. Cleft lip and palate are classified into two categories, namely, non-syndromic cleft lip with or without palate (NSCL/P) and syndromic cleft lip with or without palate (SCL/P), based on the presence of systemic symptoms. Certain genes are implicated in the development of both SCL/P and NSCL/P, with the *IRF6* gene being the most prominent [2,3]. Although significant phenotypic differences exist among various SCL/Ps, many cases are accompanied by systemic or local skeletal abnormalities [4,5]. Previous studies have focused on the mechanisms underlying the occurrence of cleft palate; however, further exploration is necessary to elucidate how abnormalities in these genes contribute to the associated skeletal phenotypic anomalies.

*Arhgap29* is one of the pathogenic genes associated with NSCL/P. It exerts its biological functions by participating in essential processes such as cell adhesion, cytoskeletal reconstruction, cell proliferation, apoptosis, and cell motility, all of which are crucial during embryonic development [6]. Existing studies have demonstrated a significant association between variants of *Arhgap29* and NSCL/P [7,8]. Additionally, the *IRF6*–*Arhgap29*–RhoA signaling pathway is reported to be involved in the craniofacial morphogenesis [9]. *Arhgap29*^K326X/+^ point mutation mice exhibited cleft palate and epithelial adhesions, which may occur between the maxillary and mandibular tooth buds, or between the palatal shelves and the tongue [10].

Our research group previously identified *Arhgap29* as a target gene through genome-wide association studies (GWAS) and whole-exome sequencing of families affected by cleft lip and palate. Subsequently, we constructed an *Arhgap29* knockout mouse model using CRISPR/Cas9 technology. The *Arhgap29*^−/−^ mice exhibited phenotypes including cleft palate, delayed palate fusion, delayed molar development, ectrodactyly, and tail deformities, which closely resemble the phenotypes associated with ectrodactyly–ectodermal dysplasia and cleft lip / palate (EEC) syndrome. The deletion of *Arhgap29* regulates the proliferation and apoptosis of palatal mesenchymal cells via the *Edar*/NF-κB pathway [11]. All these research findings highlighted the specific role of *Arhgap29* in SCL/P.

Meanwhile, *Arhgap29*^−/−^ mice exhibited bone abnormalities both throughout the body and in localized areas. It remains unclear whether these changes are secondary to the cleft palate or whether they reflect a direct role of *Arhgap29* in bone development.

The coordinated development of craniofacial organs relies on precise spatiotemporal regulation. Mandibular abnormalities are considered core initiating factors in syndromic craniofacial diseases, such as the Pierre Robin sequence [12,13]. Defects in mandibular development may subsequently lead to the posterior displacement of the tongue and the obstruction of palatal elevation, ultimately resulting in phenotypes such as cleft palate [14,15]

The role of *Arhgap29* in bone development through the aforementioned mechanisms remains unclear. In this study, we examined the involvement of *Arhgap29* in Meckel’s cartilage fusion, as well as in the formation of mandibular and skull bones, and in osteoblastic differentiation. We provided direct evidence that *Arhgap29* precisely regulates mandibular growth, thereby supporting future research aimed at developing therapeutic interventions for craniofacial anomalies.

## 2. Results

### 2.1. General Characteristics of Arhgap29 Knockout Mice

**Figure 1:**
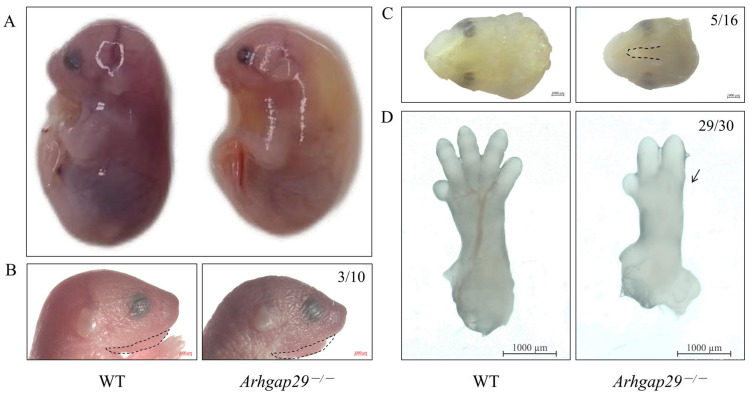
General characteristics of *Arhgap29*^−/−^ mice. (**A**) Gross image of the E15.5 mice. (**B**) Image of the head of the P0 mice (the black dashed box indicates the mandible). The penetrance of mandibular anomalies in *Arhgap29*^−/−^ mice is 30% (3/10). (**C**) Image of the palate of the E17.5 mice. The black dashed line indicates the cleft palate with 31.25% penetrance (5/16). (**D**) Image of the forelimb digits of the E14.5 mice. The black arrow indicates ectrodactyly with 96.67% penetrance (29/30).

Compared to WT mice, *Arhgap29*^−/−^ mice exhibited phenotypes including reduced body size, edema (Figure 1A), abnormal mandibular morphology (Figure 1B), cleft palate (Figure 1C), and ectrodactyly (Figure 1D).

### 2.2. The Impact of Arhgap29 on Cranial, Maxillofacial Cartilage Bones, and Digits

**Figure 2:**
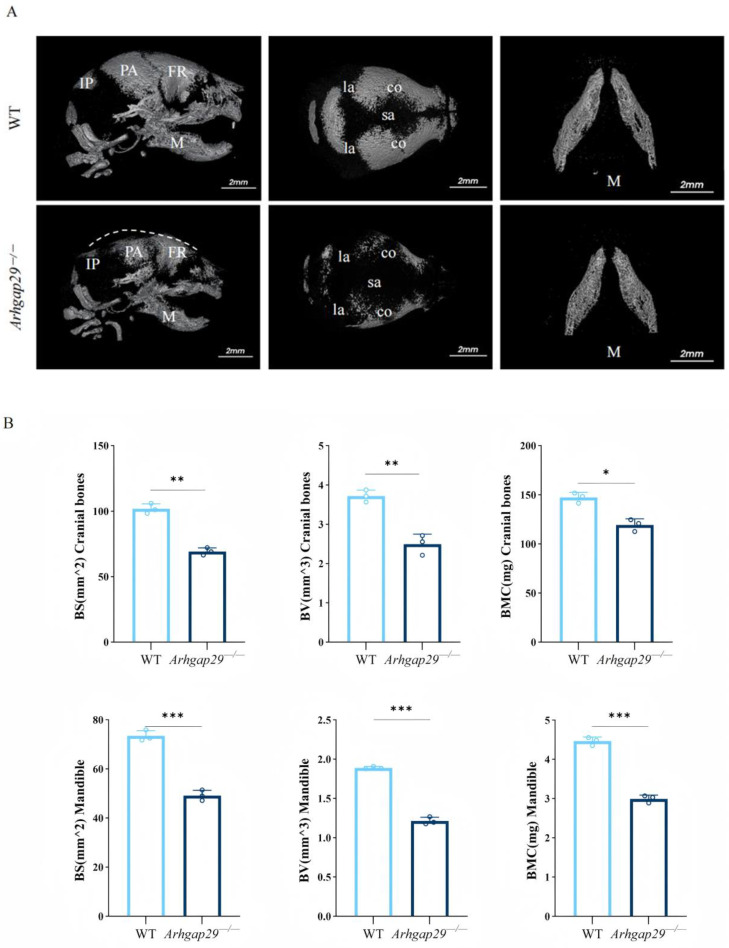
(**A**) Three-dimensional reconstruction images of micro-CT scanning of WT and *Arhgap29* deletion specimens. The white dashed line indicates the outline of the collapsed skull of the *Arhgap29*^−/−^ mice. FR, frontal bone; PA, parietal bone; IP, interparietal bone; M, mandible; co, coronal suture; sa, sagittal suture; la, lambdoidal suture. (**B**) The quantitative analysis of the cranium and mandible showed that the average bone surface area, bone volume, and bone mineral content were reduced to varying degrees in both the cranium and mandible of *Arhgap29*^−/−^ mice compared with WT mice. BV, bone volume; BS, bone surface; BMC, bone mineral content. All data: *** *p* < 0.001; ** *p* < 0.01; * *p* < 0.05.

To further clarify the impact of *Arhgap29* deficiency on craniofacial bone development, micro-computed tomography (micro-CT) scans were performed on newborn WT and *Arhgap29*^−/−^ mice. The micro-CT scans revealed that *Arhgap29*^−/−^ mice exhibited uneven bone surfaces, a collapsed skull roof, widened cranial sutures (including the coronal, sagittal, and lambdoid sutures), and a reduced mandibular volume (Figure 2A). Quantitative analysis confirmed a significant reduction in bone surface area, volume, and mineral content in *Arhgap29*^−/−^ mice (Figure 2B). These findings demonstrate the essential role of *Arhgap29* in craniofacial bone development and the maintenance of bone mass.

**Figure 3:**
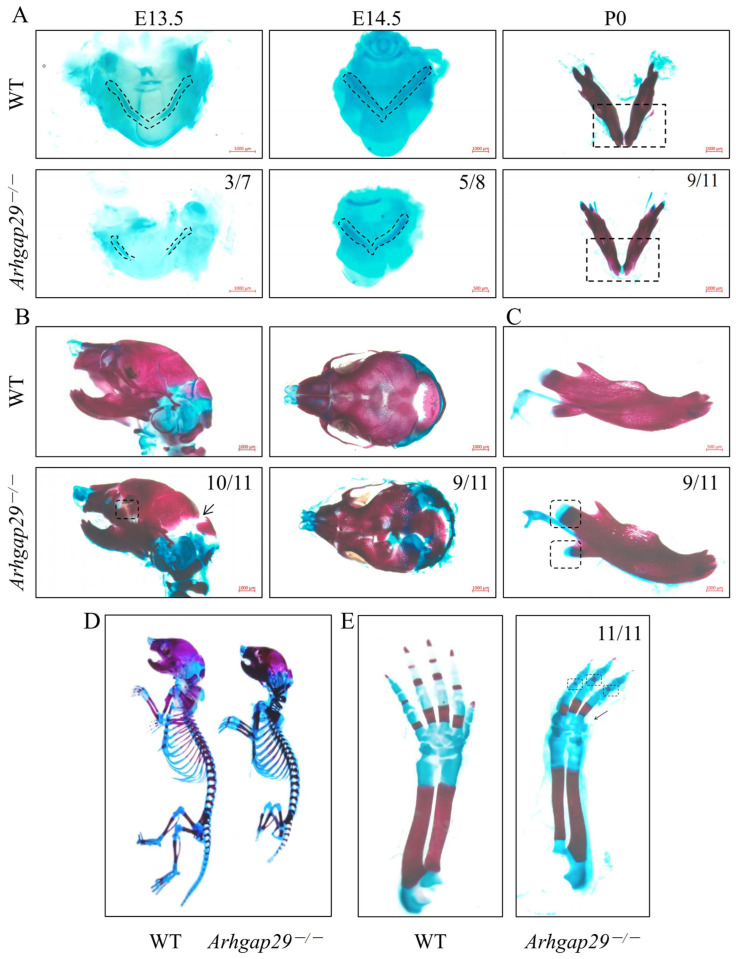
Alcian blue and Alcian blue/alizarin red staining results of cartilage and bone in the craniofacial region and limbs of WT and *Arhgap29*^−/−^ mice. (**A**) Alcian blue staining was performed on Meckel’s cartilage during three developmental stages: E13.5, E14.5, and P0. (**B**) Alcian blue/alizarin red staining of cranial and facial bones at P0. (**C**) Alcian blue/alizarin red staining of the mandible at P0. (**D**) Alcian blue/alizarin red staining of the whole skeleton of mice. (**E**) Alcian blue/alizarin red staining of the front limbs of mice; the *Arhgap29*^−/−^ mice have ectrodactyly.

The cartilage and bone in the craniomaxillofacial region and limbs of mice were observed using Alcian blue and Alcian blue/alizarin red staining methods. Our key findings include the following: Meckel’s cartilage development was notably affected; at E13.5, *Arhgap29*^−/−^ mice exhibited shorter and unfused Meckel’s cartilage. By E14.5, fusion had occurred, yet the size remained reduced. At P0, we observed a delayed degeneration compared to WT mice (Figure 3A). Furthermore, cranial defects were evident, including a wider gap in the skull-occipital bone and delayed development of the frontal-maxillary bone junction, as well as reduced mineralization of the parietal bone (Figure 3B). Mandibular defects included the delayed degeneration of the condylar and coronoid process cartilage (Figure 3C), suggesting a reduced degree of endochondral ossification. Systemic abnormalities were also noted, such as a shorter body length (Figure 3D and Figure S1) and shorter forelimbs exhibiting ectrodactyly with reduced digit ossification (Figure 3E).

### 2.3. Histomorphological Phenotypes in Meckel’s Cartilage and Mandible of Arhgap29^−/−^ Mice

**Figure 4:**
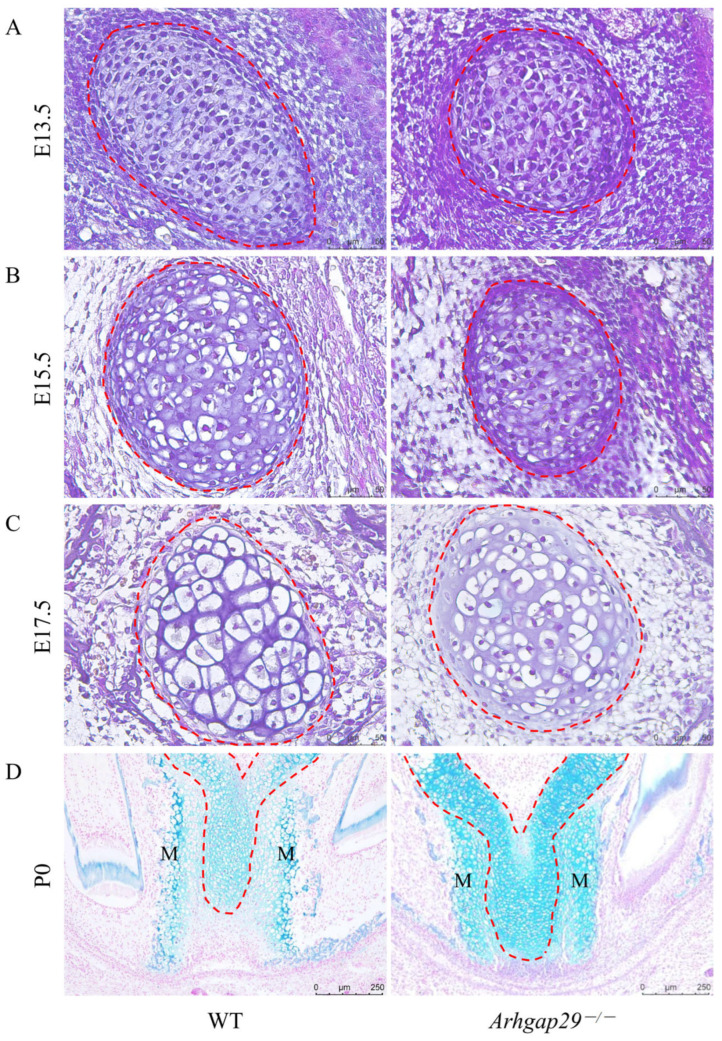
Histological characteristics of Meckel’s cartilage. The results of H&E staining of Meckel’s cartilage in E13.5 (**A**), E15.5 (**B**), and E17.5 (**C**) mice, along with Alcian blue staining of Meckel’s cartilage in P0 mice (**D**). (**A**–**C**) illustrate the coronal section staining results of the mouse head, demonstrating the delayed hypertrophy of Meckel’s chondrocytes in *Arhgap29*^−/−^ mice. (**D**) presents the axial section staining results of the mouse head, which reveal the delayed degeneration of Meckel’s cartilage. The red dashed line indicates Meckel’s cartilage (MC); the mandible is labeled as M. Three mice from each group were chosen for every time period.

At E13.5, *Arhgap29*^−/−^ mice exhibited smaller Meckel’s cartilage compared to WT mice (Figure 4A). By E15.5, Meckel’s chondrocytes in WT mice demonstrated pronounced hypertrophy, whereas *Arhgap29*^−/−^ mice displayed only mild hypertrophy (Figure 4B). At E17.5, hypertrophy in WT mice further intensified, while *Arhgap29*^−/−^ mice continued to lag behind, resembling WT mice at E15.5 (Figure 4C). At P0, Alcian blue staining illustrated a reduced number of lightly stained cells in the anterior Meckel’s cartilage of WT mice, accompanied by significant mandibular hypertrophy, indicative of active cartilage degeneration and ossification. Conversely, *Arhgap29*^−/−^ mice retained a greater number of small cartilage cells (Figure 4D), suggesting a delay in cartilage degeneration and a slower rate of ossification. 

**Figure 5:**
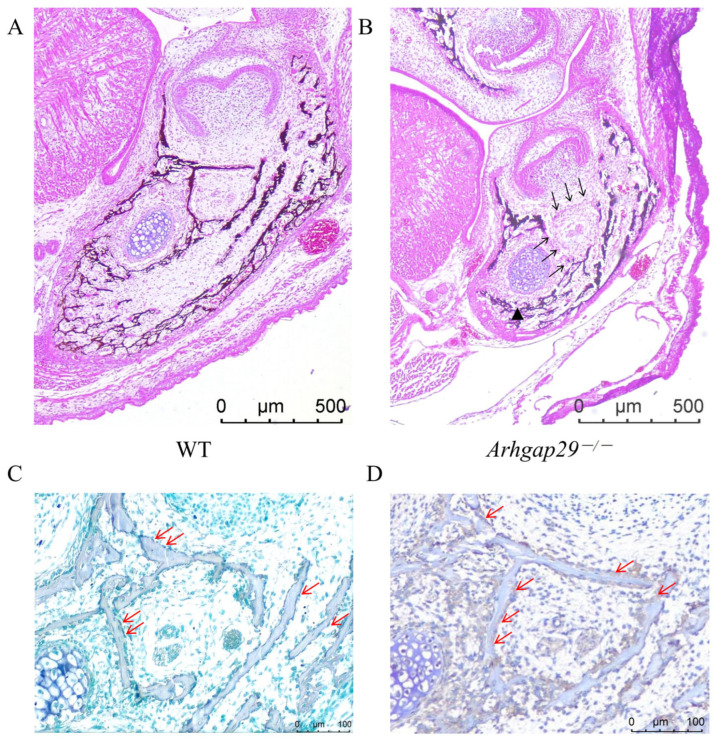
Experimental results of osteogenesis in mandibular tissue. (**A**,**B**) Von Kossa staining of mandibular tissue sections from E17.5 mice. (**C**) ALP staining of mandibular tissue sections from E17.5 WT mice. ALP-positive cells (shown in blue) are distributed on the surface of the bone matrix. (**D**) Immunohistochemical staining results of mandibular tissue sections from E17.5 WT mice. *Arhgap29*-positive cells (shown in brown-yellow) are located on the surface of the bone matrix. (The red arrow indicates osteoblasts).

Von Kossa staining was employed to evaluate bone matrix mineralization in the mandible following the deletion of *Arhgap29*. The results indicated that, compared to WT mice, *Arhgap29*^−/−^ mice exhibited a reduction in mandibular volume and a decrease in the mineralized bone matrix (as indicated by the black triangle in Figure 5B). Furthermore, the continuity of the mineralized bone matrix surrounding Meckel’s cartilage and the mandibular nerve canal was disrupted (as indicated by the black arrows in Figure 5B). To determine whether *Arhgap29* is expressed in osteoblasts, coronal head sections from E17.5 WT mice were subjected to alkaline phosphatase (ALP) staining, a marker of osteoblasts (Figure 5C), and immunohistochemistry for *Arhgap29* (Figure 5D).

### 2.4. Level of Bone Resorption in Arhgap29^−/−^ Mice Decreased

**Figure 6:**
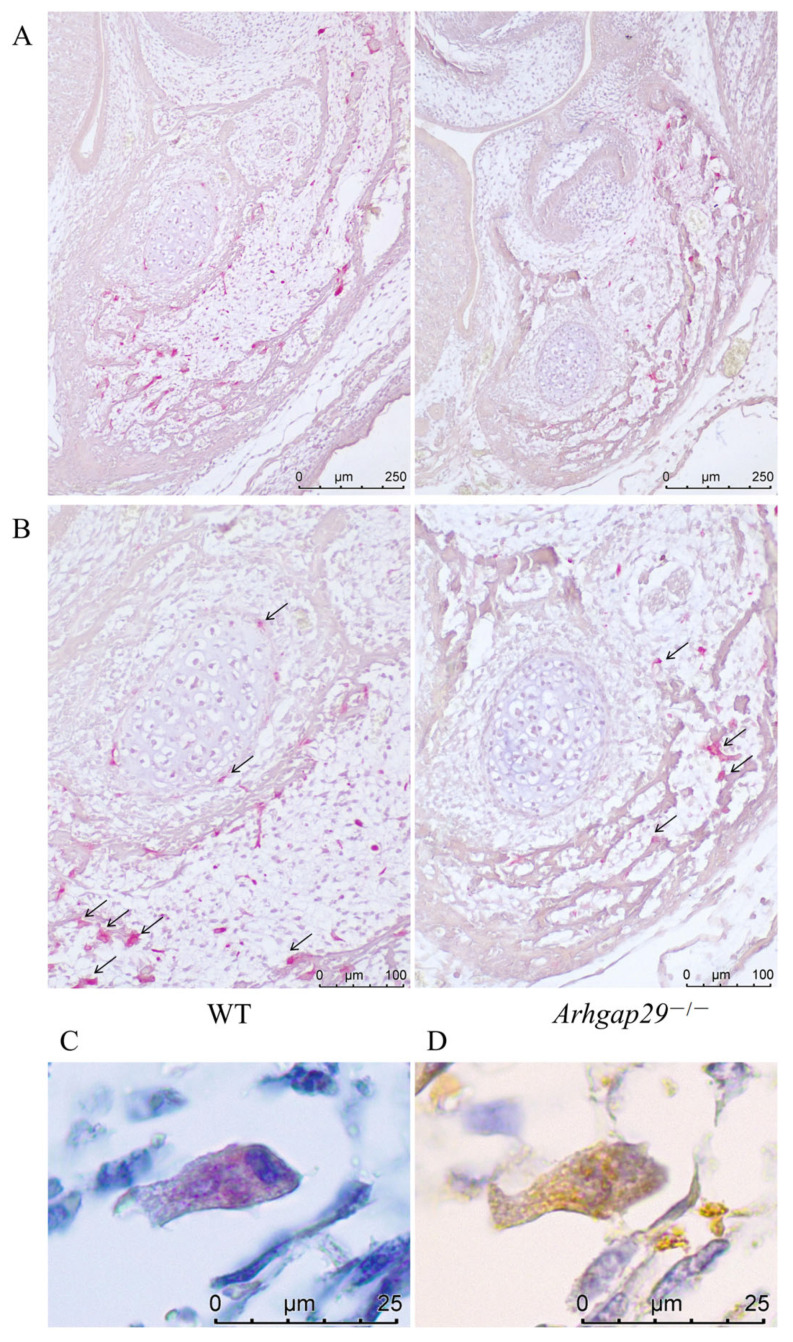
Experimental results of osteoclast activity in mouse mandibular tissue. (**A**,**B**) TRAP staining of mandibles in E17.5 WT and *Arhgap29*^−/−^ mice (osteoclasts are stained red, as indicated by the black arrows). (**C**) Staining of osteoclast marker TRAP in mandibles of WT mice at E17.5. (**D**) Immunohistochemical staining of osteoclasts in mandibles of WT mice at E17.5 (with positive cells indicated in brownish-yellow).

The TRAP staining of E17.5 mice mandible revealed that osteoclasts in WT mice were evenly distributed around the mineralized bone matrix (Figure 6A,B), indicating bone matrix resorption. In contrast, *Arhgap29*^−/−^ mice exhibited a reduced number of osteoclasts with a restricted distribution (Figure 6A,B), suggesting a diminished bone matrix resorption capacity. To determine whether *Arhgap29* is also expressed in osteoclasts of the mandible, consecutive paraffin sections of WT mice from the same developmental stage were subjected to TRAP (a marker of osteoclasts) staining and immunohistochemical staining (*Arhgap29*). The results demonstrated the co-localization of TRAP-positive cells (Figure 6C) and *Arhgap29*-positive cells (Figure 6D). These findings indicate that *Arhgap29* is expressed in both osteoblasts and osteoclasts. The loss of *Arhgap29* disrupts this balance, resulting in mandibular malformation and abnormal bone parameters in *Arhgap29*^−/−^ mice.

### 2.5. Impaired Mandibular Mineralization in Arhgap29^−/−^ Mice: Gene Expression and Pathway Alterations

**Figure 7:**
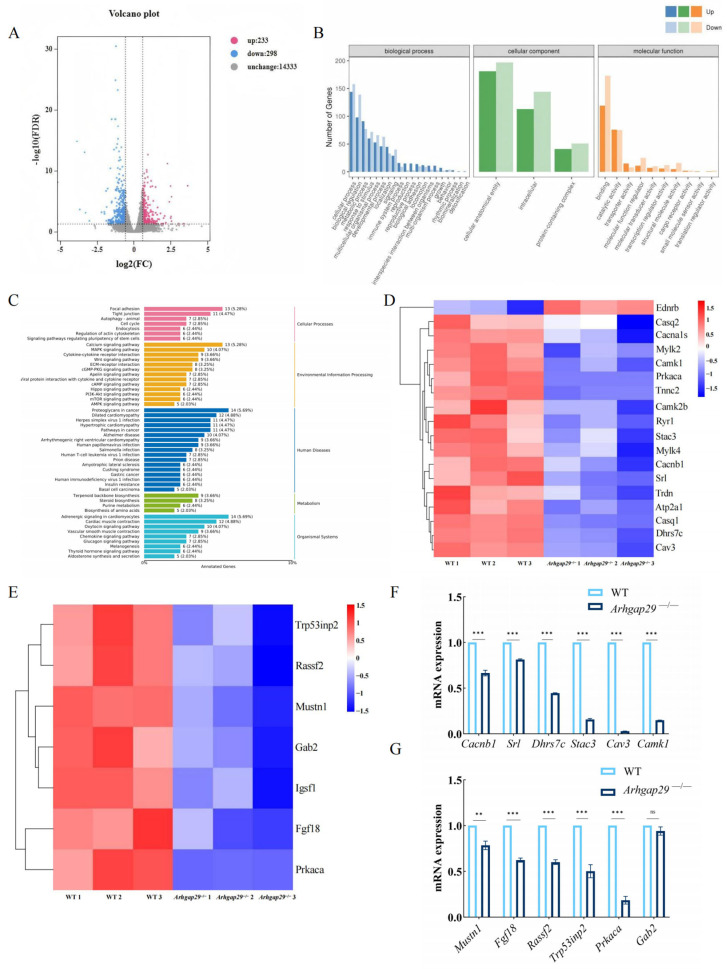
Analysis of transcriptome sequencing results for E17.5 mandibular tissue. (**A**) Volcano plot of differentially expressed genes. (**B**) GO classification annotation and enrichment analysis. (**C**) KEGG classification annotation and pathway enrichment analysis. (**D**) Heatmap of differentially expressed gene clustering in calcium signaling pathway. (**E**) Heatmap of differentially expressed gene clustering in cell differentiation. (**F**) qPCR validation of calcium signaling pathway-related molecules. (**G**) qPCR validation of cell differentiation-related molecules. (The internal reference gene used was *Gapdh*, and the relative expression of the *Arhgap29*^−/−^ group was calculated based on the gene expression levels of the WT group. Statistical analyses of differences were performed using a *t*-test. * indicates a statistically significant difference between groups. *** *p* < 0.001; ** *p* < 0.01; ns indicates no significant difference.)

To investigate the role of *Arhgap29* in mouse mandibular mineralization, mandibular tissues were collected from WT and *Arhgap29*^−/−^ mice at E17.5 for RNA-Seq analysis. Our analysis identified 531 differentially expressed genes, with 233 upregulated and 298 downregulated (|log2FC| > 1, *p* < 0.05) (Figure 7A). Gene Ontology (GO) enrichment analysis indicated significant alterations in genes associated with binding, catalytic activity, metabolism, and development (Figure 7B). Additionally, Kyoto Encyclopedia of Genes and Genomes (KEGG) pathway analysis demonstrated the downregulation of genes involved in bone development, particularly those associated with calcium, MAPK, and cAMP signaling pathways (Figure 7C). Cluster analysis further confirmed a reduced expression of key molecules in calcium signaling and cell differentiation pathways (Figure 7D,E), suggesting that the loss of *Arhgap29* impairs bone mineralization and mandible formation. Quantitative PCR (qPCR) validation revealed a significant downregulation of *Mustn1*, *Fgf8*, *Rassf2*, *Prkaca*, *Cacnb1*, and *Cav3* (genes linked to osteoblast and chondrocyte differentiation as well as calcium transport) in *Arhgap29*^−/−^ mice (Figure 7F,G). We hypothesize that the knockout of *Arhgap29* reduces the expression of mineralization-related genes (*Alpl*, *Ibsp*, and *Ocn*) by disrupting cellular differentiation and calcium signaling, ultimately impairing mandibular osteogenesis. 

### 2.6. Decreased Transcriptional Levels of Bone Development-Related Molecules in Digits of Arhgap29^−/−^ Mice

**Figure 8:**
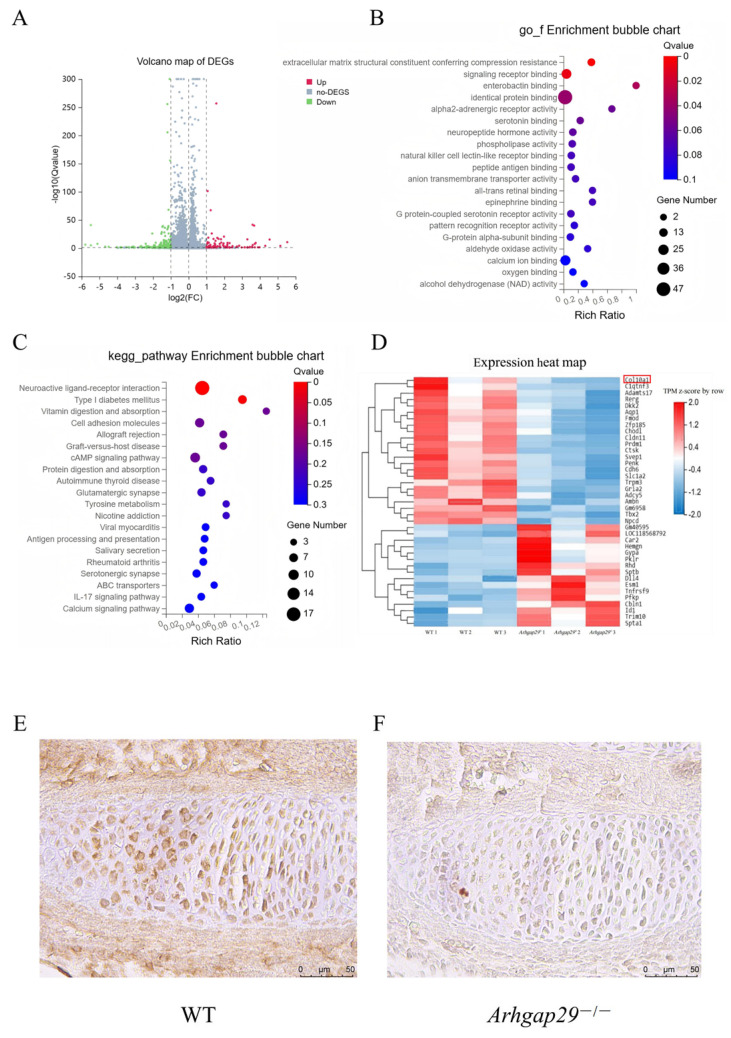
Analysis of E13.5 digit transcriptome sequencing results. (**A**) Volcano plot of differentially expressed genes. (**B**) GO classification annotation and enrichment analysis. (**C**) KEGG classification annotation and pathway enrichment analysis. (**D**) Heatmap of differentially expressed gene clustering (inside the red line frame is *Col10a1*). (**E**,**F**) E13.5 digit COL10A1 immunohistochemical staining results (positive cells appear brownish-yellow).

To further clarify the impact of *Arhgap29* on digit development, we performed RNA-seq on E13.5 mouse forelimb digits. We identified 310 differentially expressed genes (92 upregulated and 218 downregulated; |LogFC|≥1, *p* ≤ 0.05) (Figure 8A). The results of the GO biological function enrichment analysis revealed that the differentially expressed genes were predominantly enriched in 20 molecular function categories, including extracellular matrix structural constituent, signaling receptor binding, and enterobactin binding. The cellular component enrichment analysis of the differentially expressed genes indicated that they were mainly enriched in 20 categories, such as extracellular region, extracellular matrix, collagen trimer, collagen-containing extracellular matrix, and cell surface (Figure 8B). The KEGG pathway enrichment analysis results showed that the differentially expressed genes were enriched in several signaling pathways, including neuroactive ligand–receptor interaction, vitamin digestion and absorption, cAMP signaling pathway, protein digestion and absorption, and calcium signaling pathway (Figure 8C). Notably, collagen gene *Col10a1* was downregulated in the protein digestion and absorption pathway (Figure 8D).

Given that *Col10a1* is essential for chondrocyte differentiation and osteogenesis [16], we confirmed the reduced expression of COL10A1 protein in *Arhgap29*^−/−^ digit chondrocytes through immunohistochemistry (Figure 8E,F). These findings suggest that *Arhgap29* regulates digit development and endochondral ossification through *Col10a1* modulation.

### 2.7. Knocking out Arhgap29 in Calvarial Cells Decreases Osteoblast Differentiation and Mineralization

**Figure 9:**
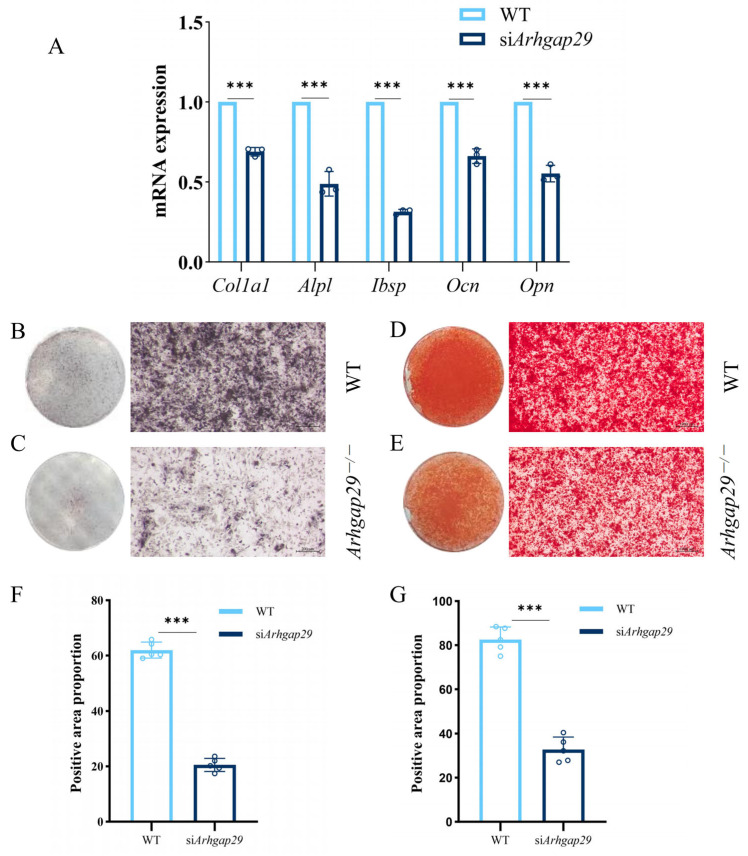
In vitro cell experiment results. (**A**) qPCR assay for osteoblast markers in cells 3 days after they were induced to differentiate. (**B**,**C**) ALP staining of WT and si*Arhgap29* cells 7 days after they were induced to differentiate. (**D**,**E**) ARS staining of WT and si*Arhgap29* cells after they were induced to differentiate for 14 days. (**F**) Quantitative analysis of alkaline phosphatase staining in cells (*n* = 5). (**G**) Quantitative analysis of alizarin red staining in cells (*n* = 5). All data: *** *p* < 0.001.

To further investigate the autonomous role of *Arhgap29* in bone development and mineralization, calvarial primary cells were isolated from newborn WT mice and induced to differentiate. Given the perinatal lethality of the majority of *Arhgap29*^−/−^ mice, *Arhgap29* expression was suppressed in WT cells using small interfering RNA (siRNA). A quantitative polymerase chain reaction (qPCR) analysis conducted three days post-induction revealed a significant reduction in the expression of osteoblast markers, including *Col1a1*, *Alpl*, *Ibsp*, *Ocn*, and *Opn*, in siRNA-treated cells (Figure 9A). Alkaline phosphatase (ALP) staining indicated smaller and less dense positive areas in the siRNA-treated cells (Figure 9B,C). Alizarin Red S (ARS) staining illustrated sparse mineralization nodules in siRNA-treated cells, contrasting with the dense and uniform staining observed in WT cells (Figure 9D,E). Quantitative analysis of ALP and ARS staining in the cells revealed statistically significant differences (Figure 9F,G). 

### 2.8. Impact of Arhgap29 Knockout on Early Mandibular Development in Mice

**Figure 10:**
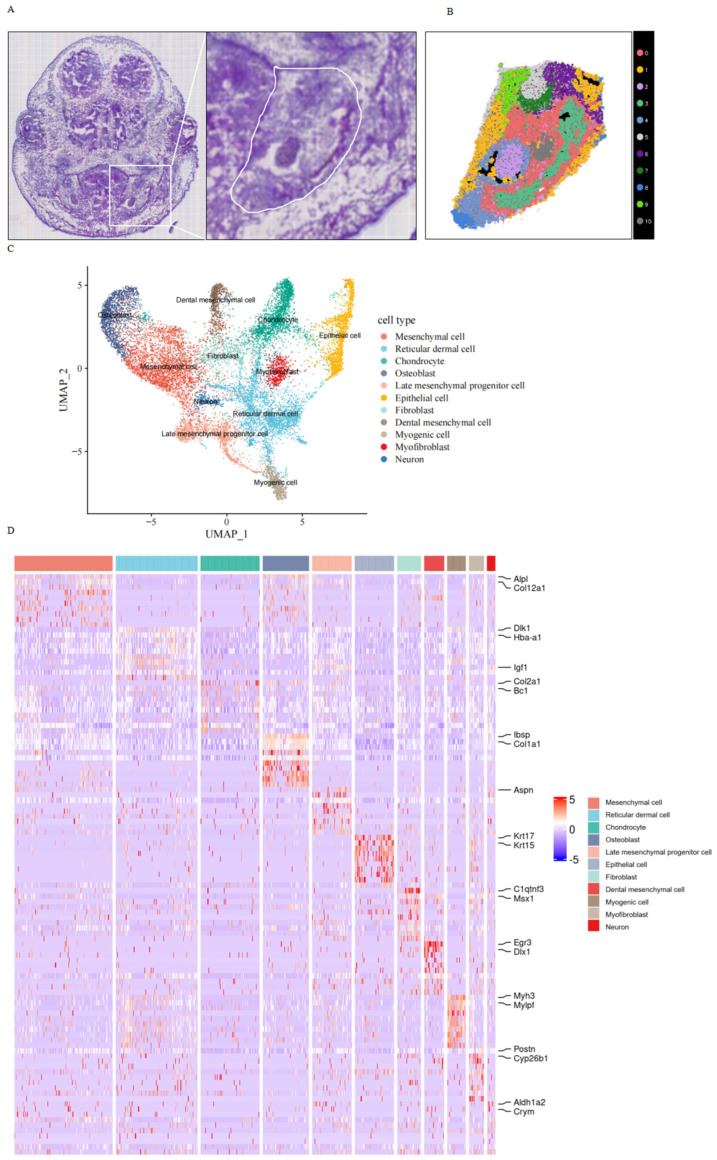

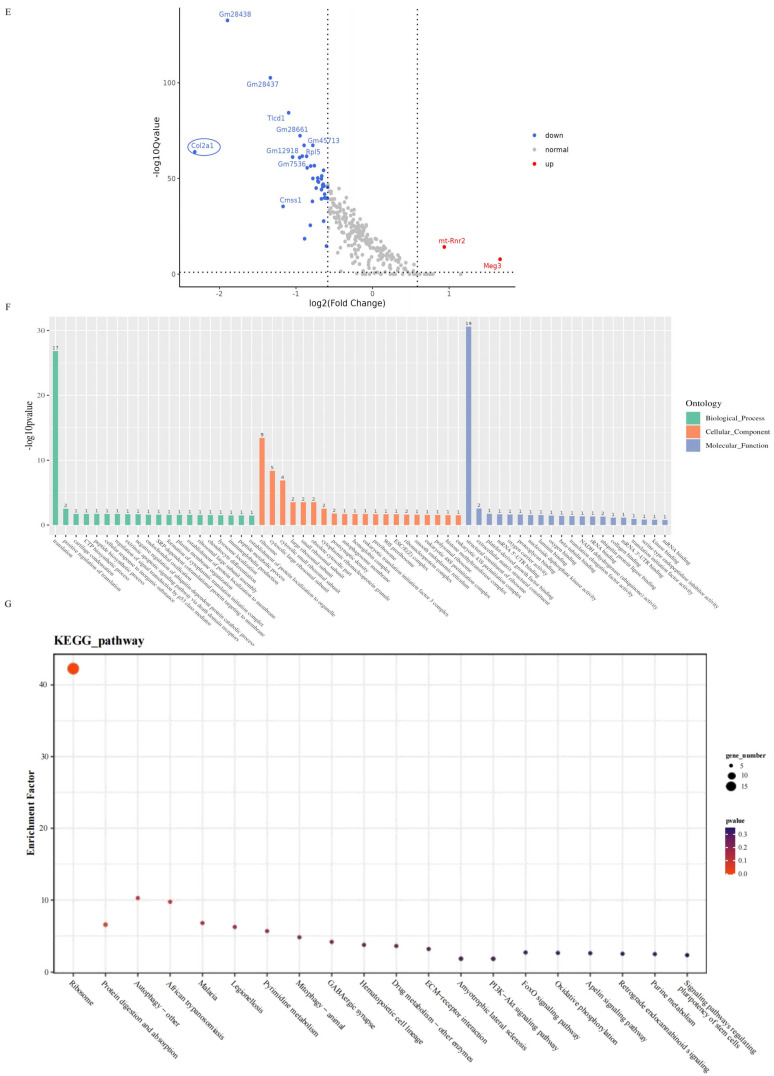

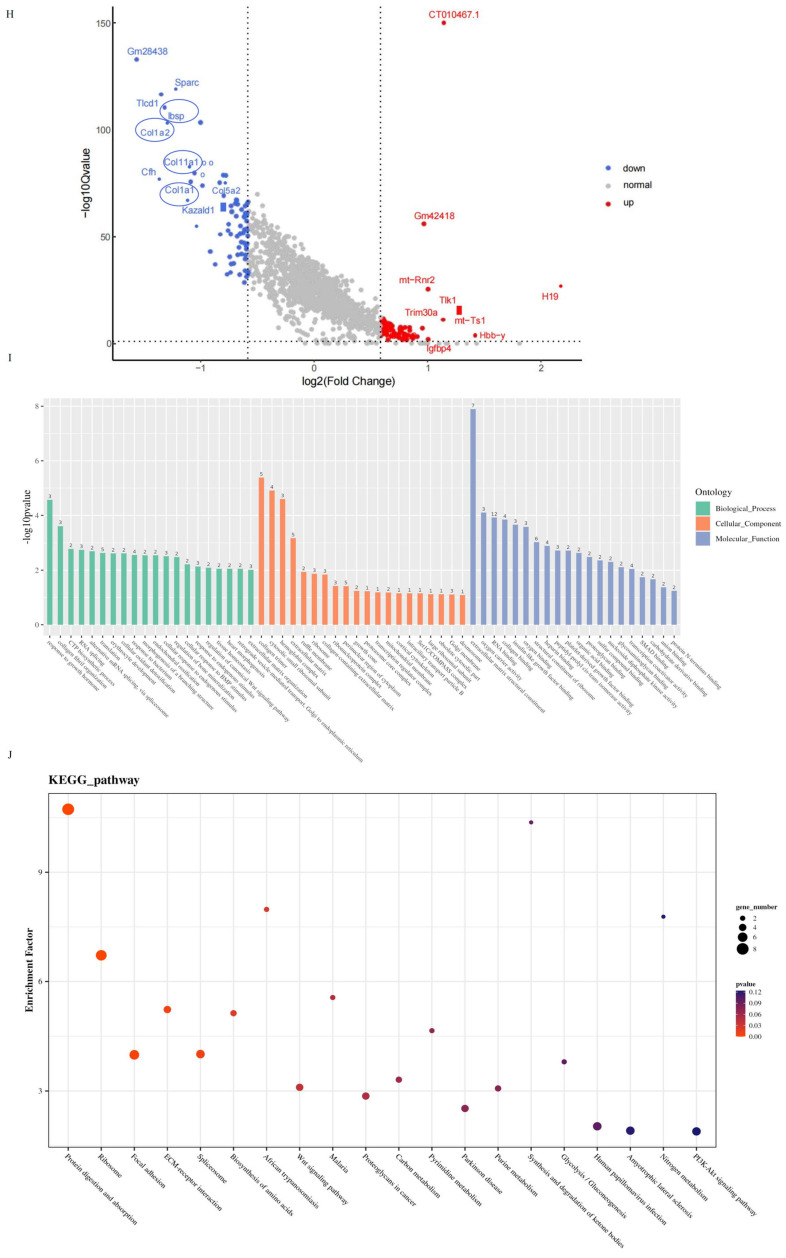
Spatial transcriptomic analysis results of mouse mandibular tissue at E14.5. (**A**) H&E staining results of the coronal section of the E14.5 mouse head. (**B**) Various cell clusters in the mandibular region. (**C**) UMAP plot of each cell cluster. (**D**) Heatmap of clustering for each cell group. (**E**) Volcano plot of the chondrocyte cluster. (**F**) GO enrichment plot of the chondrocyte cluster. (**G**) KEGG plot of the chondrocyte cluster. (**H**) Volcano plot of the osteoblast cluster. (**I**) GO enrichment plot of the osteoblast cluster. (**J**) KEGG plot of the osteoblast cluster.

The mineralization of the mouse mandible commences at E15.5. To investigate the role of *Arhgap29* in early development, we conducted spatial transcriptomics on heads at E14.5 (Figure 10A), followed by dimensionality reduction clustering, regional division of mandibular bone space (Figure 10B), differential gene expression analysis, and functional enrichment. Our analysis identified 11 distinct cell clusters (Figure 10B–D), with significant findings in mesenchymal cells, Meckel’s cartilage chondrocytes, and osteoblasts. Furthermore, GO and KEGG enrichment analyses revealed a decreased expression of ribosomal subunit genes (Figure 10F,G), which are critical for the process of translation. Given that ribosomal RNA (rRNA) transcription is frequently associated with craniofacial defects, we hypothesize that the knockout of *Arhgap29* impairs the translation of *Col2a1*, resulting in decreased expression levels.

In the osteoblast cluster, the knockout of *Arhgap29* resulted in a significant reduction in the expression of genes associated with bone formation, including *Col1a1*, *Col1a2*, *Ibsp*, and *Col11a1* (Figure 10H), as well as ribosomal subunit genes (Figure 10I,J). GO and KEGG enrichment analyses indicated that these alterations are linked to impaired RNA and collagen binding. We hypothesize that the deficiency of *Arhgap29* disrupts ribosome assembly, thereby suppressing the translation of osteogenic mRNAs and compromising the development of Meckel’s cartilage and mandibular osteogenesis; these ribosome-related defects are likely responsible for the observed mineralization deficits in *Arhgap29*^−/−^ mice.

## 3. Discussion

Syndromic cleft lip and palate (SCL/P) is frequently associated with skeletal abnormalities, such as Pierre Robin sequence (PRS), which is characterized by mandibular hypoplasia and cleft palate [17]. Embryological evidence indicates that both the palate and mandible derive from cranial neural crest cells (CNCCs), which migrate directionally to the first branchial arch and differentiate into the maxillary and mandibular processes [18,19]. The abnormal development of the mandible results in glossoptosis due to mechanical compression, thereby hindering the fusion of the palatal shelves. The variety of skeletal phenotypes observed in SCL/P, including ectrodactyly in EEC syndrome, further emphasizes the need for cross-organ developmental research [20].

*Arhgap29* is located at 1p22.1 and contains 24 exons. It encodes Rho-GTPase activating protein 29. By regulating small GTP-binding proteins, such as RhoA, it participates in biological functions including cell adhesion, cytoskeleton remodeling, cell proliferation, apoptosis, and cell movement. These functions are crucial during embryonic development [6]. *Arhgap29* is a member of the ARHGAP family; members of this family share similar functions and can compensate for one another [21]. *Arhgap6*, a closely related RhoGAP, is upregulated in the bone tissue of *Arhgap28*-null fetal mice, indicating that both *Arhgap6* and *Arhgap28* are involved in the formation of mouse embryonic bone and exhibit a certain degree of functional compensation [22]. The aforementioned research indicates that *Arhgap29* may be involved in bone development or the processes associated with bone lesions through its interaction with other genes. This provides preliminary evidence supporting the role of *Arhgap29* in bone development.

This study investigates the role of *Arhgap29* in the development of cranial and limb bones during embryogenesis. The data presented in this study comprise the first report that suggests a direct role of *Arhgap29* in the fusion of Meckel’s cartilage, as well as in intramembranous bone formation, endochondral bone formation, and mineralization. Throughout the research process, we employed general phenotypic assessments of *Arhgap29*^−/−^ mice, micro-CT imaging, histological analysis, and transcriptomic evaluation. Our findings reveal that *Arhgap29* deficiency results in the delayed fusion of Meckel’s cartilage, impaired osteoblast and osteoclast activity, reduced bone quality, and ectrodactyly. These insights into the regulatory role of Rho-GTPase-activating protein in craniofacial development enhance our understanding of the complex functions of *Arhgap29* in bone homeostasis more broadly.

The initiation of skeletogenesis begins with the migration of mesenchymal cells to the sites designated for future bones. These cells form condensations of high cellular density that outline the shape and size of the developing bones [23]. Within the condensations, mesenchymal cells either differentiate into chondrocytes to form cartilage models (anlagen) of future bones, a process known as endochondral bone formation, or they differentiate into osteoblasts, which directly form bone through intramembranous bone formation [24].

Chondrocytes undergo a series of stages, including proliferation, maturation, hypertrophy, and apoptosis, ultimately leaving behind cartilage remnants that serve as a scaffold for osteoblasts to deposit bone [25]. Current understanding suggests that the condyle and coronoid process of the mandible, as well as the anterior part of the mandible and the digits, are formed through endochondral ossification [26,27]. In contrast, the fundamental process of intramembranous ossification involves the proliferation and differentiation of mesenchymal stem cells into osteoblasts, which deposit collagen I-containing osteoid that subsequently becomes mineralized to form bone [28]. Currently, it is believed that the cranial bones and the middle part of the mandible are formed through intramembranous ossification [26].

The physical structure of Meckel’s cartilage exhibits significant alterations, which is one of the principal findings of this study. The developmental mandible partially derives from Meckel’s cartilage, which serves as a critical signaling nexus during growth [29]. During ossification, cartilage typically undergoes degradation, facilitating robust endochondral bone development in both the anterior and posterior sections of the mandible [10]. In the present study, at embryonic days 13.5 (E13.5) and 14.5 (E14.5), Meckel’s cartilage from *Arhgap29*^−/−^ mice exhibited delayed fusion and reduced length. These alterations could impair the spatial cue signaling that is crucial for the recruitment of osteoprogenitor cells and the subsequent process of ossification [30]. The condylar and coronoid processes exhibit a reduced degree of ossification in *Arhgap29*^−/−^ mice, indicating a delayed ossification process. The micro-CT findings indicate a reduction in bone volume and a depression of the cranial vaults, which corroborate the observed mechanical instability of the jaw. The deficiency of *Arhgap29* negatively impacts the normal development of Meckel’s cartilage and disrupts the process of endochondral ossification in the mandible.

In *Arhgap29*^−/−^ mice, our findings revealed further defects in intramembranous ossification, as evidenced by the abnormal unions observed between the parietal and occipital bones, as well as between the maxilla and frontal bone. These results indicate that *Arhgap29* regulates multiple stages of flat bone development, including mesenchymal condensation and osteoblast differentiation [31].

Regardless of the form of bone formation, osteoblasts, which secrete the bone matrix, and osteoclasts, which absorb it, play crucial roles in bone development, working in concert to regulate bone mass [32,33]. Our immunochemical laboratory studies demonstrated the localization of *Arhgap29* to both osteoblasts and osteoclasts, reinforcing the hypothesis that *Arhgap29* regulates the cellular components of the bone microenvironment.

Research on knockout subjects revealed significant reductions in bone mineral content, alongside alterations in bone surface area measurements and bone dimensions. This observed phenotype indicates impaired bone formation and decreased efficiency in bone matrix remodeling. The analysis of bone-forming osteoblasts derived from *Arhgap29*^−/−^ mice or engineered with suppressed *Arhgap29* expression demonstrated reduced alkaline phosphatase activity and diminished mineral nodule development under culture conditions. These in vitro findings were consistent with in vivo results, which showed decreased von Kossa-positive staining in the mandible region. Additionally, a reduction in osteoclast abundance and impeded bone resorption contributed to a complex bone phenotype. Given that the maintenance of proper bone structure relies on the coordinated activity of both osteoblasts and osteoclasts, disruptions in either cell population lead to compromised bone architecture.

Transcriptomic data analysis yields deeper mechanistic insights. In *Arhgap29*^−/−^ mice, signaling pathways critical for bone development demonstrated reduced activity due to the downregulation of calcium, Wnt, and MAPK signaling. Osteoblast function is significantly reliant on calcium signaling, which regulates proliferation, matrix mineralization, and differentiation [34]. Furthermore, the downstream non-canonical Wnt signaling pathway is crucial for bone mineralization. MAPK signaling, in response to extracellular stimuli, orchestrates gene expression changes that facilitate both osteogenesis and chondrogenesis [35]. The observed decrease in the expression of *Mustn1*, *Fgf18*, and *Prkaca* genes further supports the hypothesis that *Arhgap29* deficiency disrupts multiple cellular interaction networks. The downregulation of genes associated with cAMP signaling underscores the convergence of diverse molecular networks in *Arhgap29*-mediated regulation during normal skeletal development [36,37]. It is speculated that *Arhgap29* influences the bone mineralization process by modulating the aforementioned pathway molecules, thereby affecting the functions of both osteoblasts and osteoclasts.

Spatial transcriptomic data from E14.5 mandibles revealed a significant decline in *Col2a1* expression within Meckel’s cartilage chondrocytes, suggesting that *Arhgap29* deficiency disrupts chondrogenic matrix synthesis [38]. While *Col2a1* is crucial for maintaining cartilage integrity, the observed reduction in its expression could ultimately lead to defects in Meckel’s cartilage morphology. The disruption of ribosome biogenesis results in congenital ribosomopathies, which are characterized by distinct clinical phenotypes, including craniofacial, axial, and limb skeletal defects [39]. The reduction in ribosomal subunits indicates an overall attenuation of protein synthesis and translational stress in cells lacking *Arhgap29*. This insufficiency in protein production, induced by translational stress, may be a critical factor impeding the synthesis of cartilage and bone matrix proteins [40,41].

In vitro condensations of calvarial cells with experimentally reduced *Arhgap29* expression exhibited similar deficits in mineralization, mirroring the observed defects in mandibular formation and mineralization. The reduced density of alizarin red-stained nodules demonstrated the critical role of *Arhgap29* in driving osteoblast differentiation. The researchers concluded that osteogenic initiation exhibits high sensitivity to *Arhgap29* expression levels based on lower mineralized focus counts at early induction stages. Observational results, coupled with transcriptomic analysis, suggest that the combined disruption of intracellular signaling pathways and decreased protein production eventually result in decreased osteoblast output.

These molecular and cellular findings reveal the functional consequences of abnormal craniofacial skeletal structures. The precise alignment of the mandible with other craniofacial bones is critical for proper mastication, occlusion, and respiratory function [42]. Impaired cartilage fusion and reduced bone mineral content can increase the risk of feeding complications and dental malalignment [43]. Furthermore, widened cranial sutures may create vulnerabilities within the cranial vault, raising concerns about potential effects on brain protection and intracranial pressure dynamics [44].

Although this study has revealed the significant role of *Arhgap29* in the development of Meckel’s cartilage and the mandible, several limitations remain. Firstly, the specific molecular mechanisms through which *Arhgap29* exerts its regulatory effects have not been fully elucidated, particularly regarding how it influences the development of bone and cartilage via the calcium signaling pathway and the Wnt signaling pathway. Secondly, the relationships between *Arhgap29*, ribosome biogenesis, and translational control merit further investigation. Determining *Arhgap29*’s direct molecular targets within osteoblasts, osteoclasts, and chondrocytes is a primary requirement for elucidating the gene’s specific regulatory pathways [45]. Furthermore, combining ChIP-seq with proteomic analysis can reveal interactions between *Arhgap29* and transcription factors, as well as other cofactors. Using viral delivery systems in conjunction with small-molecule compounds should help determine the extent to which *Arhgap29*-dependent deficits can be treated postnatally [28,46]. In future research, we will further elucidate the specifics of the *Arhgap29*-specific regulatory pathway and investigate the mechanisms underlying the loss of *Arhgap29* function and potential compensatory mechanisms. 

## 4. Materials and Methods

### 4.1. Animal Model Establishment

The mouse model employed in this research was developed as described in earlier work [11]. All mice were housed under conditions free of specific pathogens (SPF). The animal experiments were performed following the approved protocols for animal use, treatment, and euthanasia by the Medical Research Ethics Review Committee of Ningxia Medical University General Hospital (approval number: 2019-057; Approval Date: 24 January 2019). This research was aligned with the principles set forth in the Declaration of Helsinki of 1964 and its subsequent revisions.

### 4.2. Micro-CT Scanning and Analysis

Newborn mice were fixed in 4% paraformaldehyde and subsequently scanned using a micro-CT system (NEMO, Pingsheng Healthcare, Kunshan, China). The scanning parameters were as follows: 60 kV, 0.12 mA, 20 frames per second, and a pixel size of 15 μm. Data collection was performed using Cruiser software (2.0.13.2), and Recon software (2.0.13.2) was used for the image reconstruction of the craniofacial skeleton. Bone volume (BV), bone surface area (BS), and bone mineral content (BMC) were the primary outcome measures. The entire cranial vault/mandible was outlined as the ROI to obtain the tissue volume (TV), with the bone tissue within the ROI representing the BV. The BS of the target region was determined by calculating the surface area of BV pixels using the Avatar software (2.0.13.2). The TV value was then substituted into the bone standard parameters to derive the bone mineral density (BMD) value, where BMC = BMD × TV. phantom was used in this process.

### 4.3. Mouse Skeletal Staining

#### 4.3.1. Alcian Blue Staining

E13.5 and E14.5 mouse embryos were preserved in 95% ethanol for a duration of three days, followed by staining with 0.015% Alcian blue for 48 h. Subsequently, they were cleared with 0.5% potassium hydroxide (KOH). After the clearing process, the embryos underwent treatment with a series of glycerol solutions at concentrations of 20%, 50%, and 100%. A Nikon stereomicroscope was utilized to obtain images for examining and analyzing variations in cartilage structure associated with different genotypes.

The heads of each group of three mice were fixed in 4% paraformaldehyde for 48 h, followed by rinsing with tap water and dehydration through a series of graded ethanol solutions. After dehydration, the samples were embedded in paraffin. Subsequently, 4 μm thick sections were prepared and stained with Alcian blue (Solarbio, Beijing, China). The internal microstructure of Meckel’s cartilage tissues was examined microscopically using a Leica Microsystems DMI 6000 B microscope (Wetzlar, Germany).

#### 4.3.2. Alcian Blue/Alizarin Red Staining

After the neonatal mice were euthanized, their skin and organs were removed and preserved in 95% ethanol for a duration of 72 h. Following this, the specimens underwent staining with 0.015% Alcian blue for 72 h and then were treated with 0.1% alizarin red for an additional 24 h. To clear the soft tissue, a 1% KOH solution was utilized. The samples were subsequently processed through a series of graded glycerol solutions. Ultimately, images were captured with a Nikon microscope to examine and analyze the variations in bone and cartilage structures.

### 4.4. H&E Staining

The heads of 18 mice (3 mice per group at E13.5, E15.5, and E17.5 stages) were fixed in 4% paraformaldehyde for 48 h. After fixation, the specimens were rinsed with water, dehydrated through a graded series of ethanol, and subsequently embedded in paraffin. The samples were then sectioned into 4-micron-thick slices (HistoCore BIOCUT, Leica, Weztlar, Germany). These sections were stained using hematoxylin and eosin (H&E), sourced from the Nanjing Jiancheng Institute (Bioengineering, Nanjing, China). The internal microstructure of the Meckel’s cartilage region was observed using a Leica Microsystems DMI 6000 B microscope (Wetzlar, Germany).

### 4.5. Immunohistochemical Staining

The heads of three E17.5 WT mice were fixed in 4% paraformaldehyde for 48 h. Following fixation, the specimens were rinsed with water, dehydrated through a graded ethanol series, and subsequently embedded in paraffin. The samples were sectioned into 4 μm thick slices using a HistoCore BIOCUT (Leica, Germany). After dewaxing and rehydration, the sections were incubated with the *Arhgap29* primary antibody (sc-377022, Santa Cruz Biotechnology, Dallas, TX, USA) overnight at 4 °C, followed by incubation with a secondary antibody (Bioss, Beijing, China) for 30 min at 37 °C. The DAB horseradish peroxidase chromogenic kit was used for color development (Beyotime, Beijing, China), and dark brown staining was considered positive. The nuclei were stained with hematoxylin (Bioengineering, Nanjing, China). After ethanol dehydration and xylene clearing, the sections were mounted with neutral glycerol and observed under an optical microscope.

The forelimbs of three E13.5 WT mice were fixed in 4% paraformaldehyde for 48 h, and the sectioning procedures were performed as described above. After dewaxing and rehydration, the sections were incubated with the COL10A1 primary antibody (bs-0554R, Bioss, Beijing, China) overnight at 4 °C, followed by incubation with a secondary antibody (Bioss, Beijing, China) at 37 °C for 30 min. The DAB horseradish peroxidase chromogenic kit was used for color development (Beyotime, Beijing, China), and dark brown staining was considered positive. Nuclei were stained with hematoxylin (Bioengineering, Nanjing, China), and the remaining steps were conducted as previously described.

### 4.6. Von Kossa Staining

The heads of 6 E17.5 mice (WT, *n* = 3; *Arhgap29*^−/−^, *n* = 3) were fixed in 4% paraformaldehyde for 48 h. Following fixation, the specimens were rinsed with distilled water, subjected to dehydration through a graded series of ethanol, and subsequently embedded in paraffin. The samples were sectioned into 4 μm thick slices using the HistoCore BIOCUT (Leica, Weztlar, Germany). After staining the mineralized bone in the sections with a commercially available calcium salt staining kit (G3282, Solarbio, Beijing, China), the sections were counterstained with HE staining solution (Bioengineering, Nanjing, China), routinely dehydrated, cleared, and mounted with neutral balsam. The internal microstructure of the mineralized bone region was observed using a Leica Microsystems DMI 6000 B microscope (Wetzlar, Germany).

### 4.7. TRAP Staining

The heads of 6 E17.5 mice (WT, *n* = 3; *Arhgap29*^−/−^, *n* = 3) were fixed in 4% paraformaldehyde for 48 h. Following fixation, the samples were rinsed with water, dehydrated through a graded series of ethanol, and subsequently embedded in paraffin. The samples were sectioned into 4 μm thick slices using the HistoCore BIOCUT (Leica, Weztlar, Germany). After dewaxing and hydration, the sections were stained for osteoclasts using a commercially available TRAP staining kit (G1050, Servicebio, Wuhan, China), followed by nuclear counterstaining with hematoxylin (Bioengineering, Nanjing, China). The sections were routinely dehydrated, cleared, and mounted with neutral balsam. Osteoclasts were observed using a Leica Microsystems DMI 6000 B microscope (Wetzlar, Germany).

### 4.8. RNA Sequencing Analysis

A total of 6 mice (wild-type (*n* = 3); homozygous (*n* = 3)), at embryonic day 17.5, were selected for this study. Total RNA was extracted from their mandibular tissues using the Total RNA kit (Cwbio, Beijing, China). RNA quality and quantity were assessed using a NanoDrop spectrophotometer and an Agilent 2100 Bioanalyzer (Thermo Fisher Scientific, Waltham, MA, USA). A library was subsequently constructed, and transcriptome sequencing was performed. The Dr. Tom multi-omics data mining system was used for data analysis, mapping, and mining. Differential gene expression analysis was performed using HISAT2 (v2.1.0) to align clean reads to the reference genome. Gene expression was quantified using RSEM (v1.3.1). Clustering heat maps of gene expression levels across samples were generated using pheatmap (v1.0.8). GO and KEGG enrichment analyses were conducted on the differential genes using phyper, with the following criteria for significance: count > 10, *p* < 0.05, |log_2_(fold change)| > 1, and false discovery rate (FDR) < 0.05. Genes meeting these criteria were defined as significantly enriched candidate genes.

### 4.9. Spatial Transcriptome Sequencing

Spatial transcriptomic sequencing was conducted on the heads of WT and homozygous mice from the same litter at E14.5. The samples were cryopreserved and submitted to Biomark for tissue optimization, fixation, staining, and fluorescence imaging. Subsequent steps included reverse transcription, spatial library preparation, sequencing, and BST matrix analysis. Data analysis focused on the mandibular region of interest and included normalization, clustering, and marker gene screening. Marker genes were annotated using the R package Seurat (v4.0.1), and functional enrichment analysis was performed using the R package clusterProfiler (v3.18.1).

### 4.10. Gene Expression Analysis by RT-qPCR

RNA was isolated from the mandibular tissue of E17.5 mice, as well as from cells following three days of induced differentiation. The extracted RNA was utilized to synthesize complementary DNA (cDNA) with the PrimeScript™ RT Reagent Kit. The quantification of mRNA expression levels for various genes was conducted using real-time PCR. Primer specifics are detailed in Table 1. This evaluation was carried out with SYBR^®^ Premix Ex Taq™ (Takara Bio) on an ABI 7500 real-time PCR instrument (Applied Biosystems). Glyceraldehyde 3-phosphate dehydrogenase (*Gapdh*) served as an internal control for normalization based on threshold cycle (Ct) values. Three independent experiments were performed to determine the relative mRNA levels. To calculate the expression levels of the PCR products, the comparative Ct method was employed.

### 4.11. Isolation of Osteoblasts and In Vitro Differentiation

Primary calvarial osteoblasts were isolated from the calvaria of 1-day-old mouse pups. Briefly, the collected calvaria were minced and incubated with 2.5% tryptase (Gibco, 25200072, New York, NY, USA) at 37 °C with gentle agitation for 5 min. The supernatant was discarded, and type I collagenase (Gibco, 17100-017, New York, NY, USA) was added to cover the calvaria. The tissue was incubated at 37 °C with gentle agitation for 10 min. After discarding the supernatant, type I collagenase was again added to cover the calvaria, followed by incubation at 37 °C with gentle agitation for 20 min. The resulting supernatant was collected and centrifuged at 300× *g* for 5 min at room temperature. The cell pellet was resuspended in α-MEM (Gibco, 12571063, New York, NY, USA) supplemented with 10% fetal bovine serum (Gibco, 10099141 C, New York, NY, USA) and incubated under 5% CO_2_ at 37 °C overnight. On the next day, the culture medium was replaced with a fresh medium to remove non-adherent cells. Upon reaching the third passage, the standard medium was replaced with a differentiation medium (OriCell, Guangzhou, China), and the cells were cultured in the dark, with medium changes every three days.

### 4.12. ALP and Alizarin Red Staining

ALP and alizarin red staining were performed to assess the osteogenic potential of primary calvarial osteoblasts. Primary calvarial osteoblasts were induced to differentiate along an osteogenic lineage. For ALP staining, the cells were differentiated for seven days. For alizarin red staining, the cells were differentiated for 7 and 14 days. At the corresponding time points, the cells were washed three times and fixed with 4% paraformaldehyde for 20 min. Subsequently, the cells were stained for 30 min with either an ALP staining solution (Solarbio, Beijing, China) or an alizarin red S staining solution (OriCell, Guangzhou, China) at pH 8.3. Following two washes, the cells were photographed by light microscopy (TS2-S-SM, Nikon, Tokyo, Japan).

### 4.13. Statistical Analysis

Statistical analyses were performed using GraphPad Prism version 10.0 (GraphPad Software, La Jolla, CA, USA). Data were presented as mean ± SEM. Statistical significance between independent samples was determined using an unpaired, two-tailed Student’s *t*-test. A *p*-value of less than 0.05 was considered statistically significant. The levels of significance were denoted as * *p* < 0.05, ** *p* < 0.01, and *** *p* < 0.001, indicating increasingly significant differences compared to the control group.

## 5. Conclusions

Our research indicates that the knockout of *Arhgap29* significantly reduces the fusion capability of Meckel’s cartilage, delays the hypertrophic process of chondrocytes, disrupts the balance between osteoblasts and osteoclasts, causes ectrodactyly, and impairs the mineralization of craniofacial and digit bones. Mechanistic studies reveal that *Arhgap29* influences multiple pathways, including the calcium signaling pathway, the MAPK pathway, and ribosome biogenesis, suggesting that *Arhgap29* is a critical factor in maintaining craniofacial skeletal stability. These findings provide a foundation for further research into the molecular mechanisms through which *Arhgap29* regulates gene transcription and the production of extracellular matrix in various tissues, while also offering significant theoretical insights into the molecular mechanisms underlying syndromic cleft lip and palate accompanied by skeletal deformities.

## Figures and Tables

**Table 1:** Sequences of primers used for RT-qPCR.

Gene Name	Forward Primer (5′-3′)	Reverse Primer (5′-3′)
*Gapdh*	CATGTTCCAGTATGACTCCACTC	GGCCTCACCCCATTTGATGT
*Cacnb1*	TCACCTTTGAGCCCAAGGAC	GGCACGTGCTCTGTCGATTT
*Srl*	TTTCAGGACAAGCAGAGGTGG	GATTTGCCAACACTCCACGG
*Dhrs7c*	TCAGTGTGAAGACGTGAGCA	TGGCATAGAGGCTCTCCAGT
*Stac3*	TCTCAGCTGTCCACTGTCCT	CTTCAGTCGCTGTAGCCCAC
*Cav3*	TCAATGAGGACATTGTGAAGGTAGA	CAGTGTAGACAACAGGCGGT
*Camk1*	AGCAGGCGGAAGACATTAGG	TTGGGGTGCTTGATCTTGTGTGT
*Mustn1*	ATGCGGGACTACGAGCAAG	GGTTGGGGACATTGGGCATA
*Fgf18*	GATGTATTCAGCGCCCTCCG	TCCACTAGGAGCTGGGCATAC
*Rassf2*	GCACCTTATTCCCTCCACCC	CTCGTCTTCCTCCTCTCGGT
*Trp53inp2*	TCTGCTATTACTGCTCTGGCA	AGGAGCTGTATAGCTGTCCTGT
*Prkaca*	TGCAGCAGATCTTATGAGGC	TGCTTTAGCTTCACCACCTTCT
*Gab2*	CCGACTCCATCGAGCTTCTT	TGGGCTCATGGGGATGTAGA
*Col1a1*	TTCTGCCCGGAAGAATACGTATC	GGACATCTGGGAAGCAAAGTTTC
*Ibsp*	CGGCCACGCTACTTTCTTTATAA	AGTGTGGAAAGTGTGGAGTTCTC
*Alpl*	AGCTTCCTTCTGTTCGTGCT	TTGACGTTCCGATCCTGAGT
*Ocn*	GGAGGGCAATAAGGTAGTGAACA	TAGGGCAGCACAGGTCCTAAATA
*Opn*	GCCGCCTGATCAAGTTCTCC	GCTGCTGCCCACAGTAGAAA

## Data Availability

The data that support the findings of this study are available from the corresponding author upon reasonable request.

## Supplementary Materials

The following supporting information can be downloaded at: https://www.mdpi.com/article/10.3390/ijms26104647/s1.
