# Supplementary figures and images for: Arhgap29 Deficiency Directly Leads to Systemic and Craniofacial Skeletal Abnormalities
Source: Int J Mol Sci. 2025 May 13;26(10):4647. doi: 10.3390/ijms26104647 (PMC12111240; doi:10.3390/ijms26104647)

Figure S1

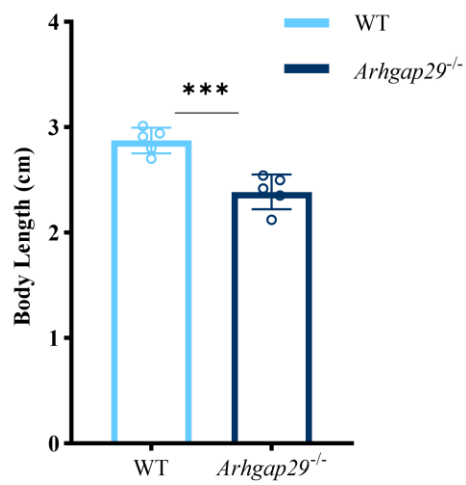

Supplement: Supplementary file 1 [file ijms-26-04647-s001.zip › ijms-3579365-supplementary.pdf]
